# The Hearst Health Prize: The First Five Years

**DOI:** 10.1089/pop.2021.0174

**Published:** 2022-08-08

**Authors:** Alexa M. Waters, Alexis Skoufalos, Emily Frelick, Gregory Dorn, David B. Nash

**Affiliations:** ^1^ Department of Family and Community Medicine, Thomas Jefferson University, Philadelphia, Pennsylvania, USA.; ^2^ College of Population Health, Thomas Jefferson University, Philadelphia, Pennsylvania, USA.; ^3^ Hearst Health, San Francisco, California, USA.

**Keywords:** population health, public health, social determinants of health, health policy, Triple Aim

## Abstract

The Hearst Health Prize is the first national annual award for excellence in population health. The prize was established “to discover, support, and showcase the work of an individual, group, organization, or institution that has successfully implemented a population health program or intervention that has made a measurable difference” in health outcomes. Now, 5 years since the award's inception, this article reflects on how submissions for the prize collectively mirror – and may even predict – changes within the field of population health. It examines how the most successful programs demonstrated genuine, measurable improvements in health outcomes and/or health behaviors. In exploring the work of these outstanding programs, the aim of this article is to help disseminate best practices, advance the mission of the prize, and inspire improvements in population health practices.

## Introduction

Over the past decade, the field of population health has evolved rapidly, both by design and of necessity. The enactment of the Affordable Care Act in 2010 and a shift toward value-based care have generated interest in work that moves the United States closer to achieving the Triple Aim^1^: improving health outcomes and quality of care, while reducing health care costs. There is growing recognition that improving the health of populations must include promoting healthy behaviors, equity in housing, education, environment, and other factors (collectively known as *social determinants*) that influence health and often fall outside the realm of traditional health care delivery.^2^ Engaging resources outside the health system is gaining new importance in population health.

More recently, the COVID-19 pandemic has taken an immense toll on our nation and the world, stressing health care systems and exposing deepening health inequalities. The pandemic also has given new and urgent importance to, and generated increased public interest in, population health efforts. Families are struggling with how best to protect their loved ones from illness, often while wrestling with challenges such as joblessness, food and housing insecurity, and access to health care. Perhaps now more than ever, innovation in approaches to population health management is essential to creating positive change in today's dynamic health care environment.

In 2015, Hearst Health, a division of the global company Hearst, entered into a collaborative partnership with the Jefferson College of Population Health (JCPH) in Philadelphia, Pennsylvania to foster population-based approaches to improving health. Together, these organizations developed objective criteria to recognize outstanding achievement in population health initiatives and created the Hearst Health Prize. The first of its kind, this national annual award was established “to discover, support, and showcase the work of an individual, group, organization, or institution that has successfully implemented a population health program or intervention that has made a measurable difference” in health outcomes.^3^ Prize creators sought to gain insight into programs generating measurable outcomes while also emphasizing the importance of disseminating best practices, sustaining progress, and advancing innovation.

Five years since the award's inception, Hearst Health has awarded $650,000 to program finalists from across the country. This article reflects on the history of the prize and how its applicants' submissions collectively mirror – and may even predict – changes within the field of population health. Common themes among past submissions are examined, along with factors contributing to finalists' success. This article also examines how the most successful programs demonstrated genuine, measurable improvements in health outcomes and/or health behaviors, beyond improvements in financial, clinical, or participation end points.^3^ In exploring the work of these outstanding population health initiatives, this article hopes to advance the mission of the Hearst Health Prize and inspire further improvements in population health practices.

## Selection Process

The Hearst Health Prize is an annual award recognizing outstanding achievements in managing or improving population health. The winner receives a $100,000 cash prize and, beginning in 2018, up to 2 additional finalists each receive a $25,000 cash prize. Importantly, this is not a grant program based on a proposed scope of work but rather recognition of program planning and execution that achieves sustained and measurable success.^3^

Population health programs submitted for consideration must be currently active (not proposed or in the planning phase) and must include measurable outcomes or preliminary findings for at least 1 year. A total of 639 submissions have been received for the prize since it was first awarded in 2016, with a minimum of 112 submissions per year. This reflects submissions received as of August 9, 2019, the submission deadline for the 2020 award. Notably, all projects thus far have been conducted before the COVID-19 pandemic. The competition was paused because of the pandemic.

Submissions are screened to ensure that they comply with all of the application rules. Those that do are reviewed and scored by JCPH faculty – 2 readers for each submission – using weighted objective criteria (Table 1). After the initial scoring, the top-rated submissions are sent for review to the Hearst Health Prize Judges, a panel consisting of 9 national leaders with expertise in diverse areas including public health, data and technology, and value-based care. Judges include policy makers, clinicians, researchers, and academicians. Each judge individually reviews the top submissions and assigns scores based on the selection criteria. Judges must recuse themselves from reviewing submissions from organizations with which they have an affiliation or relationship. The judges' scores and comments are compiled, and they convene as a group to deliberate and discuss the relative merits of each project. Together, they then consider which projects best exemplify the mission of the prize and highlight priorities for future population health endeavors. After deliberation, they have a limited time to submit any revisions to their original scores, and the highest-ranking submissions become the 3 finalists. The judges do not know which of the finalists is the ultimate winner until the public announcement.

**Table 1.** Evaluation Criteria for Submissions

Criterion	Weight
Population health impact or outcome, demonstrated by measurable improvement	30%
Use of evidence-based interventions and best practices to improve the quality of care or services provided	20%
Promotion of communication, collaboration, and engagement	20%
Scalability and sustainability	15%
Innovation	15%

## Project Characteristics

Projects submitted for the prize reflected a wide variety of models and interventions being implemented in population health across the United States. These programs often sought to go beyond a focus on acute, individual episodes of care toward more proactive, patient-centered, and community-oriented services. Although some programs were led by large health systems, others were spearheaded by community-based and nonprofit organizations. Programs varied widely in their target populations, themes of focus, and outcomes measured, and evolved in recent years in response to growing recognition of the importance of social determinants. Notably, some of these characteristics are difficult to classify and may overlap; this review is meant to simply provide an overview. Salient features of past prize submissions are examined to understand how they may reflect trends in population health initiatives across the nation.

### Geography

**FIG. 1.**
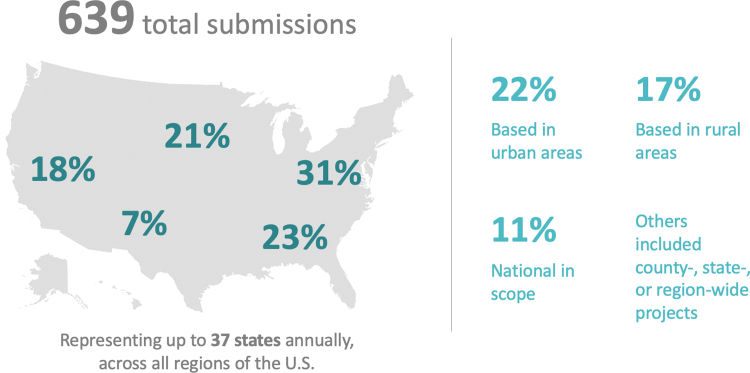
Geographic distribution of Hearst Health prize submissions, 2015–2019.

Organizations across all regions of the country submitted projects for the prize, representing up to 37 states annually (Figure 1). In the first year, nearly half of submissions were from urban areas, especially those in the Northeast; in subsequent years, there was greater representation from nonurban areas and other US regions. Projects ranged in scope from individual clinics or hospitals to nationwide efforts.

### Types of organizations

The majority of projects were conducted by health systems or hospitals, many of which had academic affiliations. A large number also were led by nonprofit organizations. Some projects, particularly those with a focus on technology or devices, were led by industry. Over time, there was increasing diversity in the types of organizations submitting projects. These included local health departments, Accountable Care Organizations, payers, employers, and even state parks.

### Health concerns addressed

Programs addressed a number of different health concerns, most commonly chronic conditions and those requiring complex care, in addition to programs promoting general well-being and preventive care. Many submissions focused on maternal, child, or adolescent health, behavioral health, diabetes, or cardiovascular diseases. Other important conditions or areas of focus included obesity, substance use (particularly opioids), food and nutrition, homelessness, cancer, family planning, and end-of-life care.

### Themes of focus

Population health interventions are inherently complex, often simultaneously addressing multiple issues that contribute to poor health. Submissions for the prize certainly reflect this complexity. In the first 2 years, the majority of projects focused on themes of care utilization and coordination, access to care, and health behaviors. A smaller number of early projects focused on screening and prevention, transforming care protocols, or improvements in quality of care outcomes (eg, reducing 30-day readmissions), using them as proxies for health outcomes. Few early projects directly addressed the impact of social determinants of health such as housing, food, or access to transportation.

Over time, there has been a gradual shift in the projects' prominent themes. Projects have increasingly turned to technology and community partnerships to better coordinate care, improve communication among stakeholders, and drive healthy behaviors.

### Target populations

Initiatives targeted a variety of populations, most commonly including children and adolescents, women and infants, and the elderly. Many of the interventions included low-income and under-resourced populations, including members of underserved minorities, and those who were uninsured or affected by issues such as substance use, food insecurity, or homelessness.

**FIG. 2.**
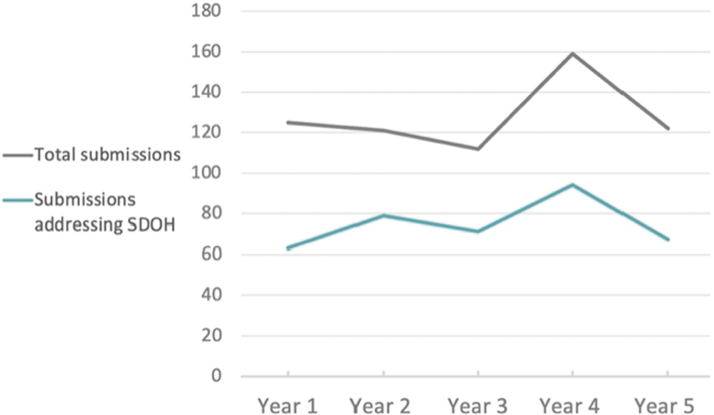
Submissions addressing social determinants of health (SDOH) each year (approximate number).

Beginning in year 2, an increase was noted in submissions that also made an effort to reduce health disparities by addressing social factors (Figure 2). More submissions included attempts to ameliorate the effects of poverty, inadequate education, food or housing insecurity, and low health literacy. This trend reflects a growing recognition across the health ecosystem that improvements in population health cannot be achieved solely through providing better medical care.

These evolving themes in the submission set reflect a central goal in population health management: to proactively address person-centered and community-oriented factors that affect health.

### Outcomes

The programs submitted for consideration measured diverse outcomes, both in improving care processes and health itself. While some focused on concrete quantitative outcomes such as reductions in mortality or emergency room visits, others focused on more difficult-to-measure qualitative aspects of wellness and prevention, including mental health or self-efficacy. The nature of the submissions has changed as the field has evolved and matured. Early on, the majority of projects pointed to cost control, utilization, and quality of care delivery as evidence of improved population health. Quality measures themselves were also broad, ranging from improvements in HIV control to decreased preterm births. In the last 3 years, more of the programs have examined vitally important social factors including housing, access to healthy nutrition, social support, employment training, and advocacy efforts as part of their population health outcome measures.

### Stakeholders and partnerships

**FIG. 3.**
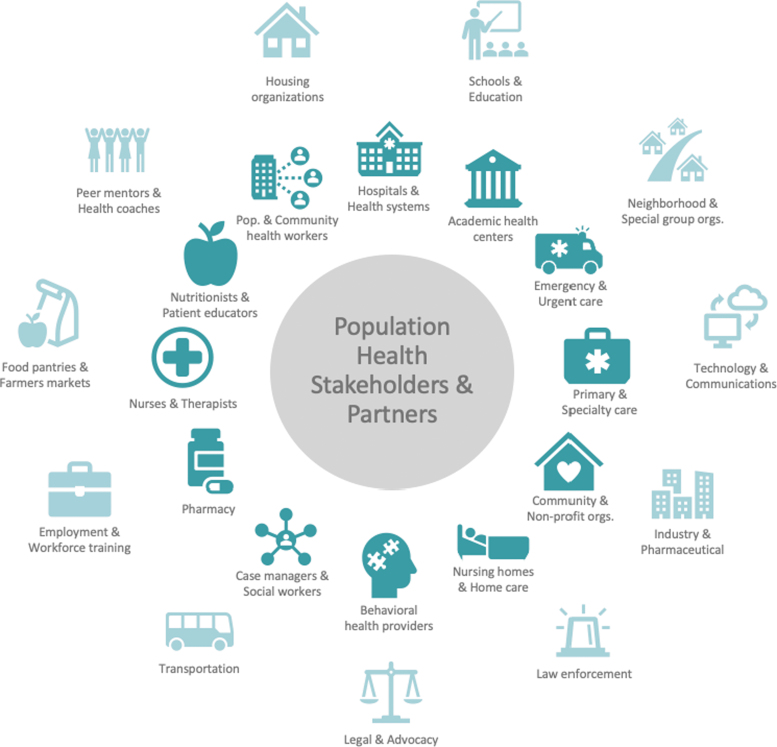
Diagram of population health stakeholders and partners, both “traditional” (inner circle) and in the larger community (outer circle).

Organizations submitting projects for the prize varied widely in size, from small community organizations to large health systems. These organizations called on a large variety of stakeholders and partners to help achieve their objectives and expand their success (Figure 3). Many projects involved new or established partnerships between “traditional” population health stakeholders, such as hospitals, primary care, and nonprofit organizations. Over time, projects increasingly involved collaborations with community partners, such as housing, government, or faith-based organizations, to lead or extend the impact of their efforts. Some also involved partnerships with industry, including pharmaceutical companies providing low- or no-cost medications and technology companies creating devices or methods for home monitoring and coordination of care.

Population health partnerships have become increasingly diverse and strategic, as stakeholders come together to address pressing health needs – of small communities to nationwide populations. Notably, data integration and technology have been important tools for facilitating such partnerships and supporting efforts to scale programs.

## Finalists: Successes, Interventions, and Evolution

Each year, 3 outstanding programs have been selected as finalists for the Hearst Health Prize (Table 2). These finalists have exemplified success in population health and demonstrated measurable improvements in health outcomes or health behaviors. Although finalists' strategies range from standard and evidence-based to innovative and novel, they shared several common features that contributed to their selection. The scope and methods of finalists' programs varied by such factors as setting, budget, and type of organization – representing a diversity of successful strategies.

**Table 2.** Hearst Health Prize Winners and Finalists, 2016–2020

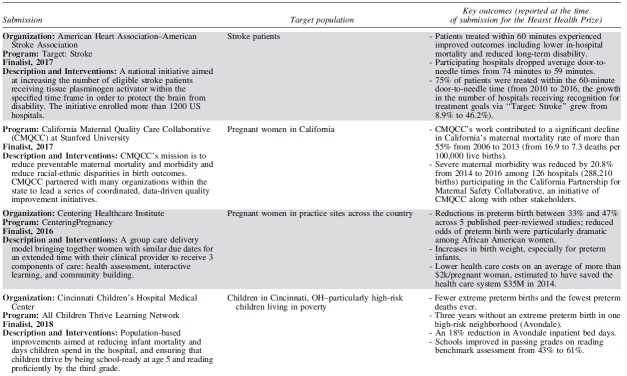 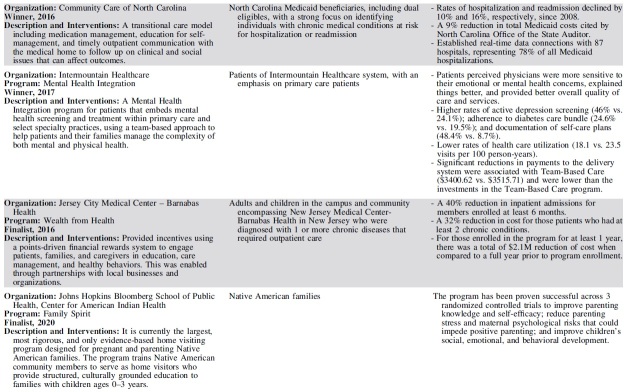 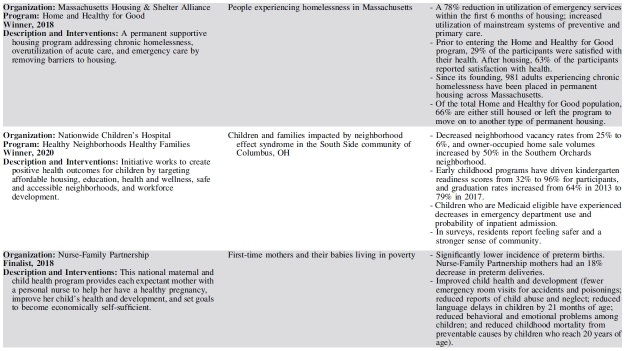 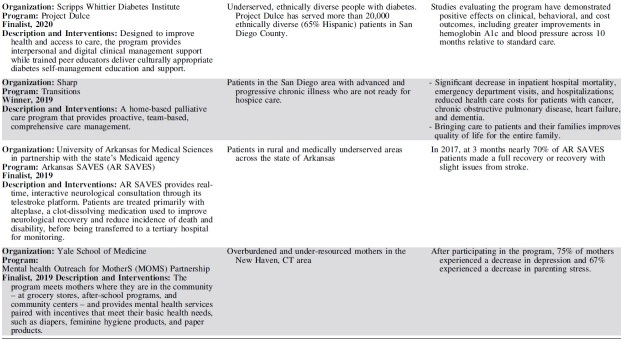

Finalists set clear, measurable goals to improve health for a specific population or community and achieved these goals by intervening through overlapping and mutually supportive interventions. The most successful programs facilitated broad communication across all stakeholder groups, often using information systems and technology to coordinate care and identify those in greatest need. Programs often sought a high level of patient and family engagement to empower self-care and promote wellness. Programs that addressed social determinants of health in combination with providing health care services often generated the broadest impacts on health, extending their efforts beyond the typical realm of health care service delivery to take on issues such as social support, housing, and education.

Prize finalists employed a variety of key intervention strategies to improve population health. The majority of finalists' interventions included efforts to coordinate and increase access to care, educate patients and families, and improve health behaviors. Many programs employed multidisciplinary or team-based care to address health concerns through several areas of expertise. Such multipronged approaches required effective communication across stakeholders, using information systems and iterative data analysis to track efforts in quality improvement. Several programs sought to disseminate evidence-based practices; for example, to improve maternity care or stroke treatment protocols. Others leveraged mental health integration, home visits, and paraprofessional outreach to patients in the community. A small group of forward-thinking finalists addressed critical social determinants by securing housing, providing career training, improving childhood education, enhancing social support, or advocating on behalf of those in need.

Finalists have evolved in their themes of focus and outcomes measured. Early finalists took on challenging tasks of coordinating complex care and reducing costs on a large scale, particularly for patients who frequently access high-cost health care services. In recent years, projects have moved beyond improving utilization and cost containment, working to demonstrate measurable improvements in morbidity and mortality and seeking to address social determinants among high-risk populations. Finalists have increasingly sought to empower patients through patient and family education and decision-making support, leading to improved patient satisfaction and self-efficacy. More recent finalists have become more inclusive in their outreach, moving beyond clinical health care delivery to involve community health workers, peer educators, and population health workers in diverse, multidisciplinary teams collaborating to improve health.

The programs chosen as the winners each year have provided models that could inspire and motivate others working to improve the health of populations.

Year 1 (2016) - Community Care of North Carolina was recognized for managing transitional care for North Carolina Medicaid beneficiaries discharged home after hospitalization. Program participants receive medication management, education for condition self-management, and timely outpatient communication with the medical home to follow up on clinical and social issues that can affect their health outcomes. Rates of hospitalization and readmission, as well as total state Medicaid costs, all decreased during the program.

Year 2 (2017) - Intermountain Healthcare's Mental Health Integration program incorporates screening and treatment for mental health issues within primary care and select specialty practices as a routine part of health care. Using a team-based approach, the program helps patients and their families manage the complexity of both mental and physical health concerns. Participants perceived improved communication and quality of care from their providers and were more likely to be screened for depression and adhere to the diabetes care bundle. The program also facilitated cost savings and lower rates of health care utilization.

Year 3 (2018) - Massachusetts Housing and Shelter Alliance (MHSA) was honored for Home and Healthy for Good, a permanent supportive housing program addressing overutilization of acute care and emergency care by removing barriers to housing. In addition to independent apartments or shared living arrangements that are integrated into the community, program participants have access to a broad range of comprehensive, community-based services, including medical and mental health care, substance abuse treatment, case management, and vocational and life skills training. Participants' use of emergency services declined, while they increased use of mainstream preventive and primary care. At the time of submission, nearly 1000 adults experiencing chronic homelessness had been placed in permanent housing across the state.

Year 4 (2019) - Sharp Transitions, part of Sharp HealthCare in San Diego, California, provides home-based palliative care for patients with advanced and progressive chronic illness who are not ready for hospice care. Bringing care to patients and their families improves quality of life for the entire family. The Transitions program has resulted in a significant decrease in inpatient hospital mortality; fewer emergency department visits and hospitalizations; and reduced health care costs for patients with cancer, chronic obstructive pulmonary disease, heart failure, and dementia.

Year 5 (2020) – Nationwide Children's Hospital's Healthy Neighborhoods, Healthy Families program works to create positive health outcomes for children by targeting affordable housing, education, health and wellness, safe and accessible neighborhoods, and workforce development. It has improved the health status and reduced unnecessary health utilization and costs for South Side neighborhood children.

## Hearst Health Prize: A Reflection of Population Health, Present and Future

The Hearst Health Prize reflects a growing focus on population health across our nation. In 2021, more attention than ever before is being paid to social determinants of health, and the prize supports the success and wisdom of programs that incorporate efforts to mitigate these social factors. No one could have predicted that a global pandemic would emerge and shine a spotlight on the health issues that previously had only been examined with flashlights. It is now widely recognized that to reduce disparities in health, we must focus on addressing social determinants, dismantling institutionalized racism, and diversifying our teams working to improve health. These bold tasks require innovation in population health efforts, reaching far beyond what the health care system alone can accomplish.

Population health initiatives, such as those submitted for the Hearst Health Prize, have been ahead of the curve in tackling these issues for many years. The sophistication of these initiatives is remarkable. Their evolution, in terms of strategies, scope, and technologies, is proceeding quickly – as it must. Unfortunately, it is not until recently that population health initiatives began to receive the attention and resources they deserve. Now, with a renewed sense of focus, we all share the responsibility to build on this momentum and harness a sense of urgency to address population health concerns.

Throughout the pandemic, the work of heroes has been celebrated. Among them are millions of essential workers at patients' bedsides and filling crucial roles across our communities. But there are unsung heroes that society has seldom recognized – those who work tirelessly to improve population health, despite all odds against them. Many of these unsung heroes are represented among the organizations that submitted applications for the Hearst Health Prize. We must continue to reward and celebrate their critical work, through broader recognition, financial support, and advocacy.

Looking to the future, we must construct a better bridge between population health and health care – to a build a system with the central focus: health and well-being.
